# Influence of Reconstitution Methods and Diluents on the Physicochemical Characteristics of Poly-L-Lactic Acid Suspensions: An In Vitro Study

**DOI:** 10.3390/polym18172044

**Published:** 2026-08-23

**Authors:** Woramate Bhorntarakcharoen, Sariya Sittiwanaruk, Wilailuck Phokai, Thanakorn Woramongkol, Thanya Techapichetvanich

**Affiliations:** Department of Dermatology, Faculty of Medicine Siriraj Hospital, Mahidol University, 2 Wanglang Road, Bangkok Noi, Bangkok 10700, Thailand; woramate.inter@gmail.com (W.B.); jenny16214@gmail.com (S.S.); wilailuckphokai@gmail.com (W.P.); thanakornwor.si@gmail.com (T.W.)

**Keywords:** poly-L-lactic acid (PLLA), commercial formulation, reconstitution, preparation protocol, visual aggregation, vertical phase separation, pilot study

## Abstract

Macroscopic and microscopic aggregation patterns of reconstituted poly-L-lactic acid (PLLA) products may vary with product formulation, diluent, preparation protocol, and time. This descriptive in vitro pilot study compared two commercial formulations, StiCol Volume (150 mg PLLA reconstituted to 9 mL) and StiCol Soft (50 mg PLLA reconstituted to 6 mL), under 11 diluent conditions and 2 composite preparation protocols: roller mixing for 30 min and manual shaking for 3 min followed by 3 h of hydration. One preparation was evaluated for each formulation–diluent–protocol combination. Gross appearance was documented immediately and at 15, 30, 45, and 60 min and 24 h; microscopy was performed at ×40 and ×100 from 15 min onward. Two evaluators applied an ordinal visual aggregation scale, and the findings were analyzed descriptively. In the observed preparations, the StiCol Soft formulation generally showed lower aggregation grades than StiCol Volume; however, the products differed in nominal PLLA concentration, particle size, and excipient composition, so this pattern cannot be attributed to concentration alone. SWFI-based conditions tended to show fewer microscopic clusters than NSS-based conditions. The roller/30 min protocol also tended to show lower grades than the manual/3 h protocol, although mixing mode and hydration duration were inseparable. Macroscopic upper-layer accumulation was interpreted as vertical phase separation with creaming/flotation-like behavior rather than sedimentation. These exploratory findings generate hypotheses for replicated studies incorporating standardized sampling, redispersibility, particle size, rheological, and injectability testing; they do not establish an administration window or improved clinical safety.

## 1. Introduction

Injectable collagen-stimulating fillers have become an important component of aesthetic dermatology for soft tissue augmentation and skin rejuvenation. Among these agents, poly-L-lactic acid (PLLA) is a biodegradable and biocompatible polymer that stimulates neocollagenesis through a gradual tissue response, resulting in progressive and sustained improvement in facial volume, skin laxity, and wrinkles [1,2,3,4]. PLLA-based fillers have been used clinically for more than two decades and are generally considered safe and effective when appropriately prepared and injected [2,4,5,6].

Unlike prefilled hyaluronic acid fillers, injectable PLLA is typically supplied as a lyophilized powder and requires reconstitution before use. The resulting suspension may vary with vehicle composition, final volume, hydration time, and mixing procedure [5,6,7]. Heterogeneous particle distribution, visually apparent clusters, or vertical phase separation may affect preparation consistency. Although uneven particle distribution has been proposed as one possible contributor to localized high particle density after injection and to papules or nodules [5,6,7], the present study did not test tissue distribution or clinical events, and this relationship remains theoretical.

Previous studies have highlighted the importance of reconstitution conditions in injectable collagen-stimulating fillers. In an in vitro study of injectable poly-D,L-lactic acid (PDLLA), Chen et al. reported that diluent type and mixing procedure were associated with differences in visual particle dispersion and aggregation [8]. Water-based conditions showed more homogeneous dispersion, whereas saline-based conditions showed more clustering; the authors proposed, but did not directly measure, a role for ionic strength and carboxymethyl cellulose (CMC) behavior [8]. These PDLLA observations provide a rationale for study but cannot be treated as mechanistic confirmation in PLLA.

Although PLLA and PDLLA are both collagen-stimulating poly-lactic acid fillers, their reconstitution behavior cannot be assumed to be identical. PLLA formulations may differ from PDLLA formulations in polymer stereochemistry, particle morphology, particle size, excipient composition, and manufacturing process [2,8,9]. Many PLLA products contain microspheres combined with excipients such as CMC and mannitol, which are intended to facilitate hydration, dispersion, and stabilization during reconstitution [2,9]. In addition, newer PLLA formulations may incorporate microsphere coating or micelle-forming technologies that are designed to improve hydrophilicity and particle dispersion during preparation [9]. These formulation-specific features may influence how PLLA particles behave under different diluent and mixing conditions.

The appearance of a reconstituted PLLA suspension may reflect interactions among particles, excipients, and the surrounding vehicle. CMC functions as a dispersing agent, and its hydration and conformation can vary with ionic strength and pH [8,10,11,12]. Mannitol and other product-specific features may also contribute [8,9,12]. Because the present study did not measure pH, osmolality, ionic strength, particle size distribution, zeta potential, viscosity, or rheology, any colloidal explanation remains hypothetical rather than an observed mechanism.

Despite the widespread clinical use of injectable PLLA, systematic visual comparisons across clinically used diluents and preparation protocols remain limited. The principal novelty of this study relative to the cited PDLLA work [8] is the structured assessment of two commercial PLLA formulations across 11 diluent conditions, 2 composite mixing/hydration protocols, and serial observations to 24 h.

This descriptive pilot study therefore aimed to compare macroscopic appearance and microscopic aggregation patterns across two commercial PLLA formulations, eleven diluent conditions, two preparation protocols that differed in both mixing mode and hydration duration, and serial observation times. We hypothesized that visually assessed aggregation and vertical phase-separation patterns would differ across these experimental conditions. The study was not designed to isolate concentration from formulation, mixing mode from hydration duration, or visual aggregation from reversible flocculation, nor was it designed to test injectability or clinical safety.

## 2. Materials and Methods

### 2.1. Study Design

**Figure 1:**
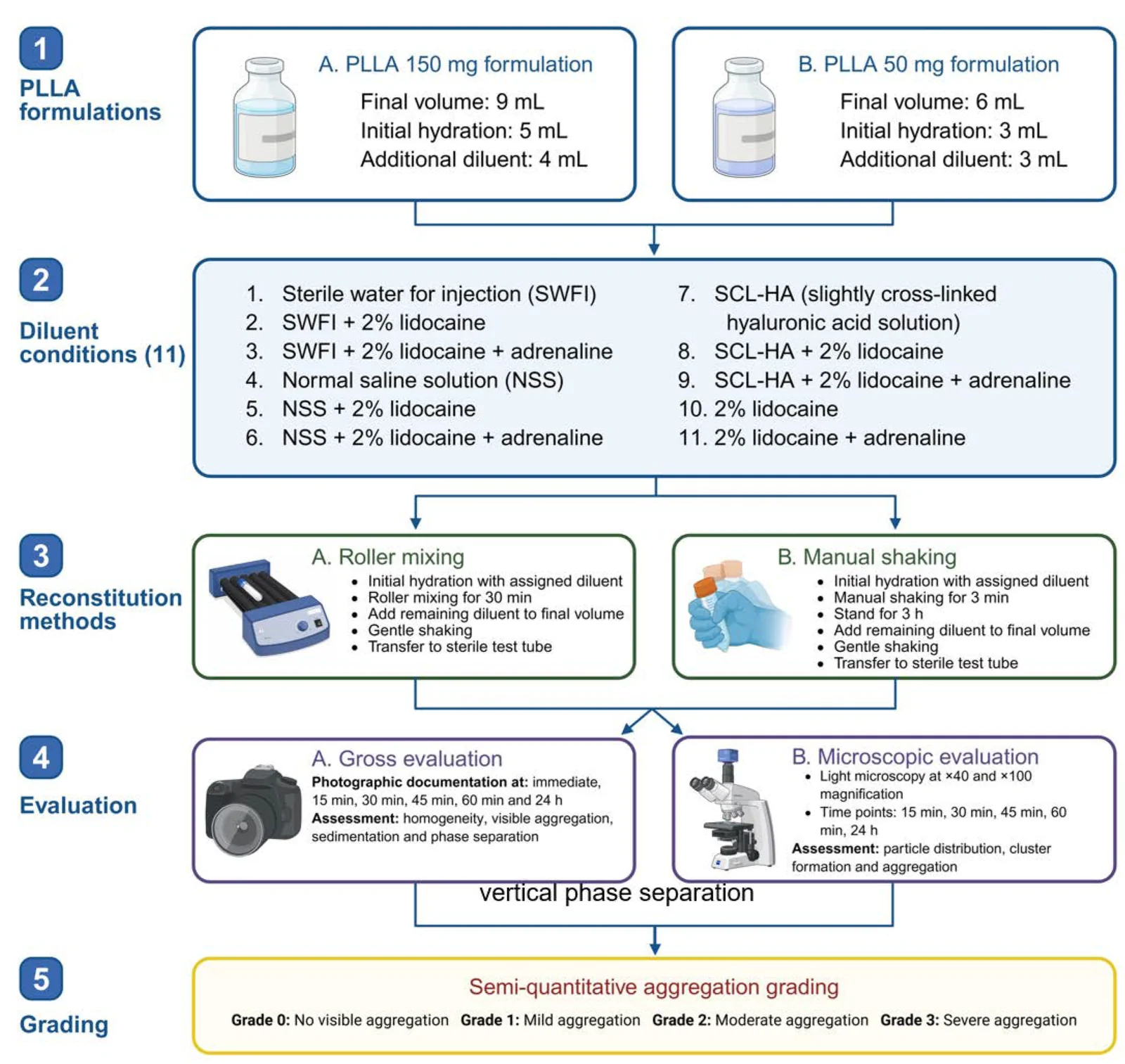
Experimental design and preparation protocols. Two commercial PLLA formulations were assessed: StiCol Volume (150 mg PLLA, final volume 9 mL) and StiCol Soft (50 mg PLLA, final volume 6 mL). Each was evaluated under 11 diluent conditions and 2 composite protocols: roller mixing for 30 min and manual shaking for 3 min followed by 3 h of hydration. Thus, mixing mode and hydration duration were confounded. Gross evaluation was performed immediately and at 15, 30, 45, and 60 min and 24 h; microscopy was performed at ×40 and ×100 from 15 min onward. One preparation was evaluated per formulation–diluent–protocol combination. Abbreviations: PLLA, poly-L-lactic acid; SWFI, sterile water for injection; NSS, normal saline solution; SCL-HA, slightly cross-linked hyaluronic acid solution.

This descriptive in vitro pilot study compared two commercial PLLA formulations under eleven diluent conditions and two composite preparation protocols. The first protocol used roller mixing for 30 min; the second used 3 min of manual shaking followed by 3 h of undisturbed hydration. Accordingly, the protocol comparison simultaneously varied mixing mode and hydration duration and cannot identify the independent effect of either factor. One independent reconstitution was prepared for each formulation–diluent–protocol combination (2 × 11 × 2 = 44 preparations), with serial observations obtained from the same tube over time and no independent or technical replicate preparations. The primary endpoint was the ordinal visual aggregation grade. Secondary descriptive observations included gross homogeneity and vertical phase separation, including upper-layer accumulation consistent with creaming/flotation-like behavior or lower-layer settling when present. These macroscopic phenomena were not assumed to represent irreversible aggregation. The overall design is summarized in Figure 1.

### 2.2. PLLA Formulations

Two distinct commercial formulations were evaluated: StiCol^®^ Volume (Contac Korea Inc., Seoul, Republic of Korea), containing 150 mg PLLA within a total lyophilized mass of approximately 365 mg and reconstituted to 9 mL (nominal PLLA concentration, 16.7 mg/mL), and StiCol^®^ Soft (Contac Korea Inc., Seoul, Republic of Korea), containing 50 mg PLLA within a total lyophilized mass of approximately 121.7 mg and reconstituted to 6 mL (nominal PLLA concentration, 8.3 mg/mL). Manufacturer information describes approximate particle sizes of 50 µm and 30 µm, respectively, and identifies CMC and mannitol as excipients [9]. Product-specific quantitative excipient composition and full particle size distributions were not available in the archived study records. Because the products differed in PLLA loading, final concentration, particle size, and potentially the relative excipient composition and manufacturing characteristics, comparisons are described as formulation comparisons and are not attributed to PLLA concentration alone.

### 2.3. Diluents

Eleven diluent conditions were evaluated: SWFI; SWFI with 2% lidocaine; SWFI with 2% lidocaine and adrenaline; NSS; NSS with 2% lidocaine; NSS with 2% lidocaine and adrenaline; 1% slightly cross-linked hyaluronic acid solution (SCL-HA; SS-A Additive Solution, Contac Korea Inc., Seoul, Republic of Korea); SCL-HA with 2% lidocaine; SCL-HA with 2% lidocaine and adrenaline; 2% lidocaine alone; and 2% lidocaine with adrenaline. For mixed base-vehicle/anesthetic conditions, 1 mL of the anesthetic stock replaced 1 mL of the base vehicle, yielding final lidocaine concentrations of 0.22% in the 9 mL preparations and 0.33% in the 6 mL preparations. In the anesthetic-only conditions, the named anesthetic solution constituted the full final volume. Component volumes are detailed in Table 1. The exact adrenaline stock concentration and preservative composition were not retained in the archived records; pH and osmolality were not measured.

### 2.4. Reconstitution Protocol

Each formulation was prepared using two composite protocols. These protocols differed in both mechanical mixing and total hydration time and are therefore termed the roller/30 min protocol and the manual/3 h protocol rather than isolated reconstitution methods. No additional arm reproduced a separate current-manufacturer reference procedure.

For the roller/30 min protocol, 5 mL of the assigned base vehicle was first added to the StiCol Volume vial and mixed on a roller mixer for 30 min. The remaining components were then added in the order shown in Table 1 to achieve 9 mL, followed by gentle shaking and transfer to a sterile, capped test tube. For StiCol Soft, 3 mL was added initially and roller mixed for 30 min; the remaining components were then added to achieve 6 mL, followed by gentle shaking and transfer.

For the manual/3 h protocol, 5 mL was first added to the StiCol Volume vial, which was manually shaken for 3 min and then left undisturbed for 3 h. The remaining components were added to achieve 9 mL, followed by gentle shaking and transfer to a sterile capped test tube. For StiCol Soft, 3 mL was added initially, followed by 3 min of manual shaking and 3 h of undisturbed hydration; the remaining components were then added to achieve 6 mL, followed by gentle shaking and transfer. Thus, any between-protocol difference may reflect mixing mode, hydration duration, or both.

### 2.5. Gross Evaluation

Gross evaluation was performed using serial photographs of the same capped test tube immediately after transfer and at 15, 30, 45, and 60 min and 24 h. Tubes were not remixed before photography. Images were acquired against a dark background with a fixed viewing arrangement; the camera model and acquisition settings were not retained in the archived records, and no post hoc exposure normalization was applied. Macroscopic assessment distinguished visible clusters from vertical phase separation. Because accumulated material was frequently located toward the upper suspension interface, this pattern was described as creaming/flotation-like behavior rather than sedimentation. The preparations were kept capped, protected from direct light, and undisturbed at ambient laboratory temperature (approximately 20–25 °C) until the 24 h assessment; the temperature was not continuously logged, and samples were not remixed before evaluation.

### 2.6. Microscopic Evaluation

Microscopic evaluation was performed on one glass slide prepared from each tube at each recorded time point (15, 30, 45, and 60 min and 24 h) without remixing the tube. Samples were examined by light microscopy at original magnifications of ×40 and ×100. The archived protocol did not prospectively standardize sampling height, drop volume, or the number and location of fields scanned before representative image capture; microscope and camera models and pixel-to-distance calibration were not retained. Consequently, accurate scale bars could not be reconstructed retrospectively. Microscopic assessment focused on particle distribution, interparticle separation, and visually apparent clusters.

### 2.7. Semi-Quantitative Aggregation Grading System

A semi-quantitative visual aggregation scale was used to describe microscopic clustering and overall heterogeneity: Grade 0, no visible clusters and a homogeneous distribution; Grade 1, small clusters with most particles dispersed; Grade 2, multiple clusters with reduced interparticle separation; and Grade 3, large compact clusters with marked heterogeneity. Macroscopic vertical phase separation was documented separately and was not used as evidence that clusters were irreversible. The scale was adapted from visual approaches used for injectable biomaterials [8] and is shown in Table 2.

### 2.8. Image Assessment

Two evaluators independently rated the available gross and microscopic image sets using the predefined ordinal criteria. They were blinded to each other’s initial ratings but not fully blinded to the experimental condition. Evaluator 1’s rating is reported outside parentheses; when Evaluator 2 assigned a different rating, that rating is reported in parentheses. A single value indicates agreement between evaluators. Hyphenated values (e.g., 1–2 or 2–3) denote a borderline or intermediate grade assigned by an evaluator and do not represent a range between the two evaluators. Disagreements were retained rather than adjudicated to a consensus score. Image fields were selected to illustrate the predominant appearance, but the field-selection process was not randomized.

### 2.9. Data Analysis

All comparisons among formulations, diluent conditions, composite preparation protocols, and time points were descriptive. Ordinal grades and macroscopic patterns were summarized without treating serial observations from the same tube as independent replicates. For interobserver agreement, each paired condition–time image rating was treated as the unit of agreement; this is distinct from treating serial observations as independent experimental replicates, and the resulting agreement statistics were used descriptively only. Interobserver agreement across the 220 paired condition–time ratings was summarized using exact agreement and linearly weighted Cohen’s kappa, with the seven ordered categories (0, 0–1, 1, 1–2, 2, 2–3, and 3) treated as successive levels. Exact agreement was 89.5% (197/220), and linearly weighted Cohen’s kappa was 0.91. No inferential comparison of experimental conditions was performed because there was one independent preparation per condition and the sampling and image-selection procedures did not support estimation of within-condition variability. Results are therefore hypothesis-generating and are reported using associative rather than causal language. Statistical analyses were performed using IBM SPSS Statistics version 29.0 (IBM Corp., Armonk, NY, USA).

## 3. Results

### 3.1. Overall Findings

Across the recorded image sets, visual aggregation grades and macroscopic separation patterns differed across commercial formulations, diluent conditions, composite preparation protocols, and observation times. StiCol Soft generally had lower grades than StiCol Volume, but this comparison reflects the combined differences between the two products rather than PLLA concentration alone. SWFI-based conditions tended to have lower grades than NSS-based conditions. The roller/30 min protocol tended to have lower grades than the manual/3 h protocol, although mechanical mixing and hydration duration were confounded. Table 3 summarizes the two evaluators’ 15 min ratings; serial descriptive data are reported in Supplementary Table S1. The 15 min time point was selected only because it was the earliest microscopic observation, not because it represents a validated administration window.

Evaluator 1’s rating is shown outside parentheses; when Evaluator 2 differed, the second rating is shown in parentheses. A single value indicates agreement. A hyphenated value (e.g., 1–2 or 2–3) denotes a borderline or intermediate grade assigned by an evaluator, not a span between evaluators. Disagreements were retained rather than adjudicated. Across all 220 paired condition–time ratings in Supplementary Table S1, exact agreement was 89.5% (197/220) and linearly weighted Cohen’s kappa was 0.91. Lower grades indicate fewer or smaller visible clusters. No inferential comparison of experimental conditions was performed.

### 3.2. Gross Appearance

**Figure 2:**
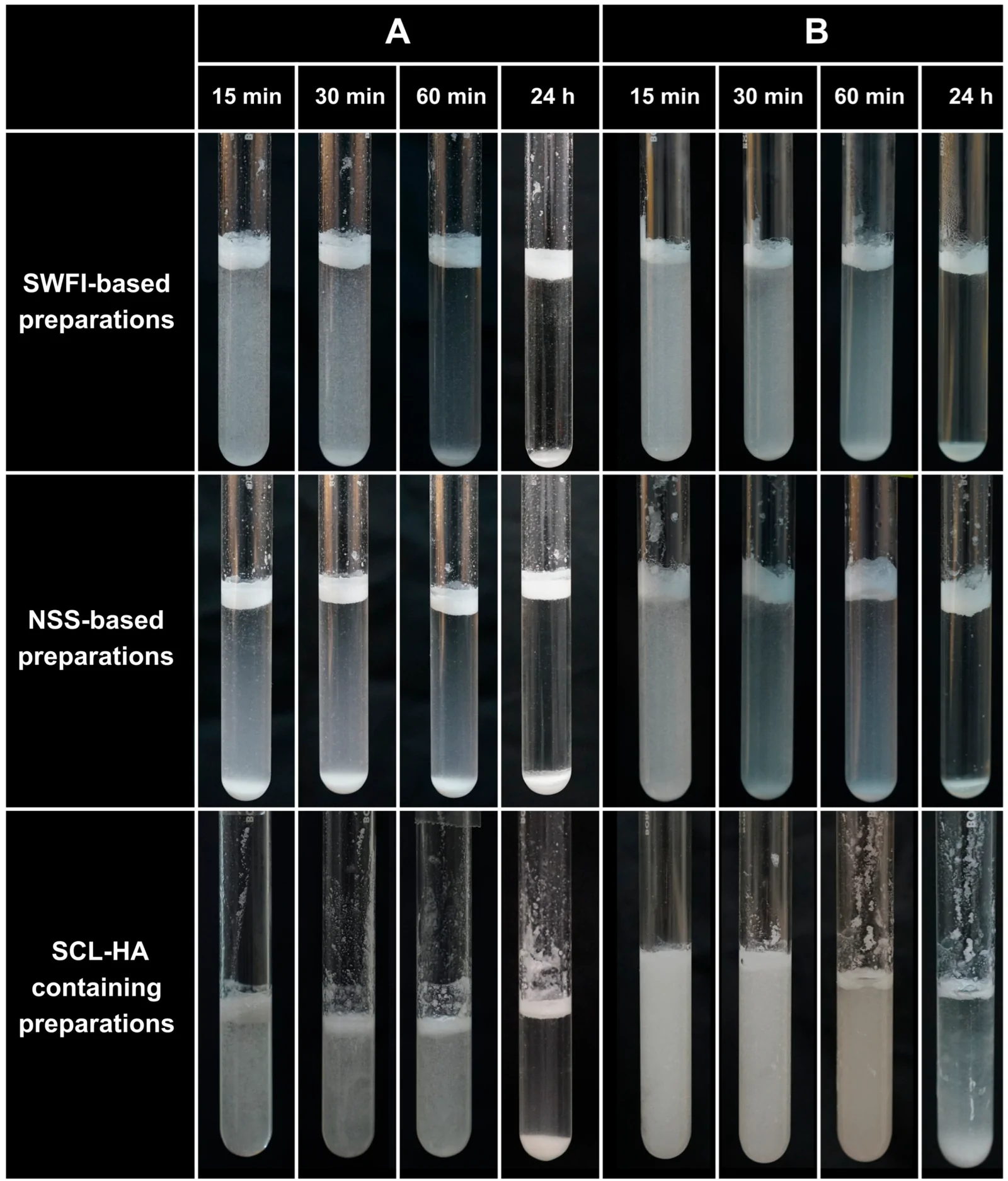

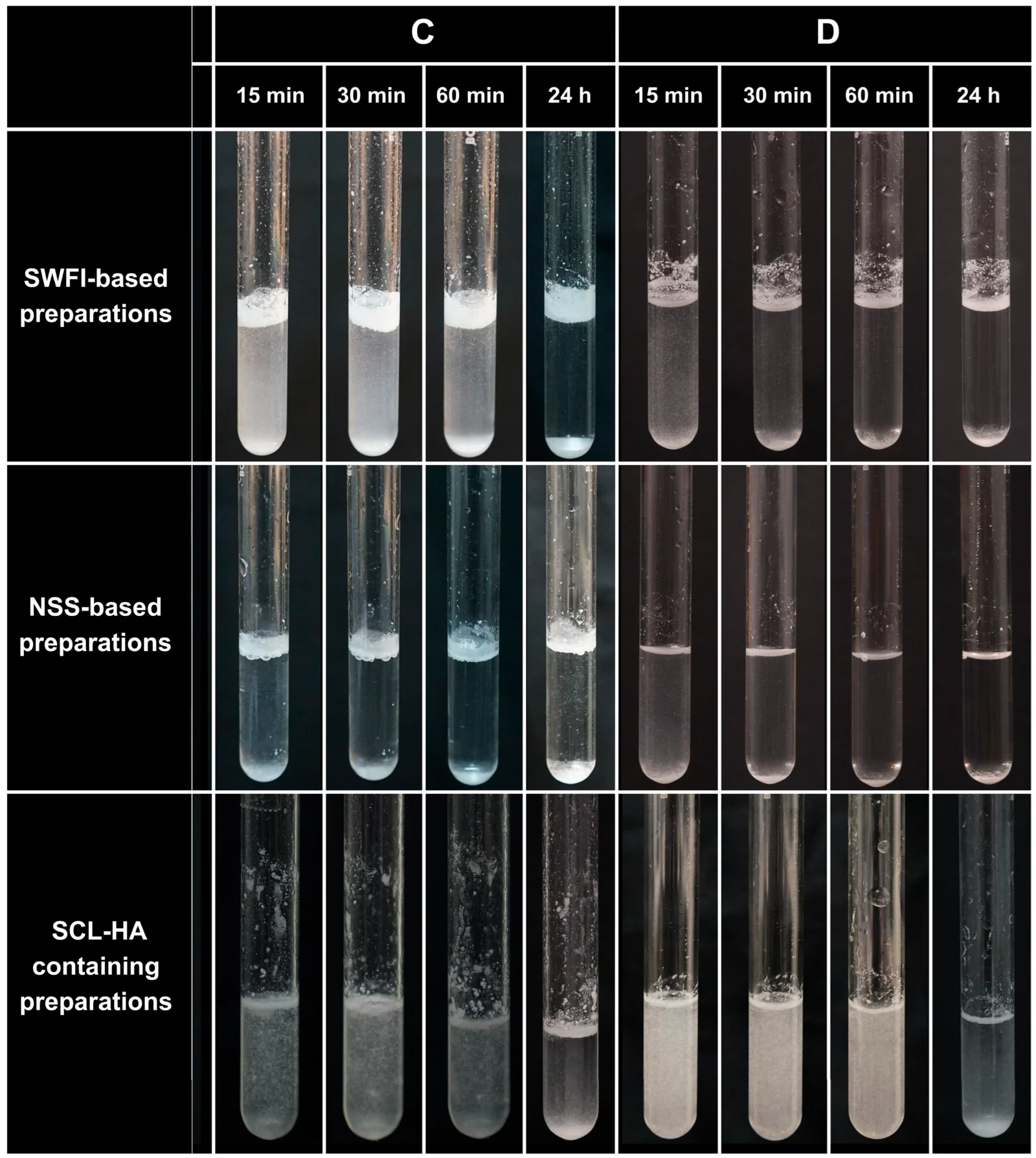
Representative gross appearance of PLLA preparations over time. The figure has been divided into enlarged panels (**A**,**B**) (StiCol Volume) and (**C**,**D**) (StiCol Soft) to improve readability. (**A**,**C**) show the roller/30 min protocol; (**B**,**D**) show the manual/3 h protocol. Rows show representative SWFI-based, NSS-based, and SCL-HA-containing conditions at 15, 30, and 60 min and 24 h. Upper-layer accumulation in many panels is described as creaming/flotation-like vertical phase separation rather than sedimentation. Images were not digitally normalized for exposure because opacity and phase appearance were observational outcomes. Abbreviations: PLLA, poly-L-lactic acid; SWFI, sterile water for injection; NSS, normal saline solution; SCL-HA, slightly cross-linked hyaluronic acid solution.

Representative gross photographs showed differences in opacity, uniformity, visible clusters, and vertical phase separation across conditions and time (Figure 2). SWFI-based image sets often appeared comparatively uniform during early observation, particularly for StiCol Soft and the roller/30 min protocol. However, an upper concentrated layer was visible in several panels, including SWFI-based conditions. Its location near the top of the liquid column is more consistent with creaming/flotation-like separation than with settling.

NSS-based image sets generally showed earlier and more pronounced heterogeneity and vertical separation than SWFI-based sets. These patterns were visually more evident for StiCol Volume and the manual/3 h protocol. Because redispersibility was not tested, the macroscopic separation cannot be interpreted as irreversible aggregation.

SCL-HA-containing image sets showed different gross separation patterns from NSS-based sets, while anesthetic-only conditions appeared intermediate in the available photographs. These qualitative descriptions are not presented as mechanistic or statistically confirmed differences.

Vertical phase separation and loss of uniformity were more apparent in several preparations from 30 min onward and at 24 h. Depending on the condition, concentrated material was located at the upper interface, within the suspension column, or near the bottom. Accordingly, the neutral term vertical phase separation is used unless direction could be determined from the image.

### 3.3. Microscopic Findings

**Figure 3:**
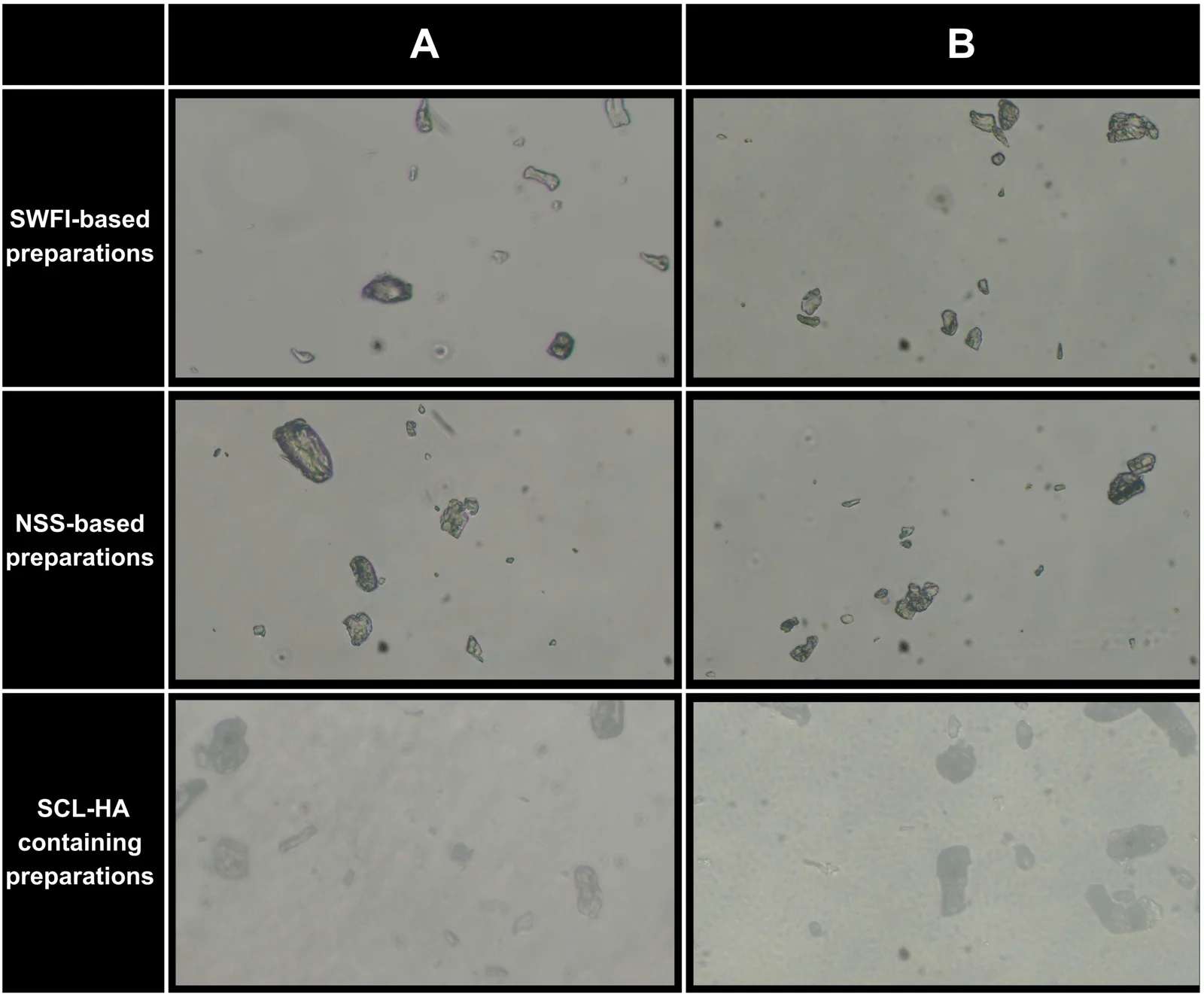

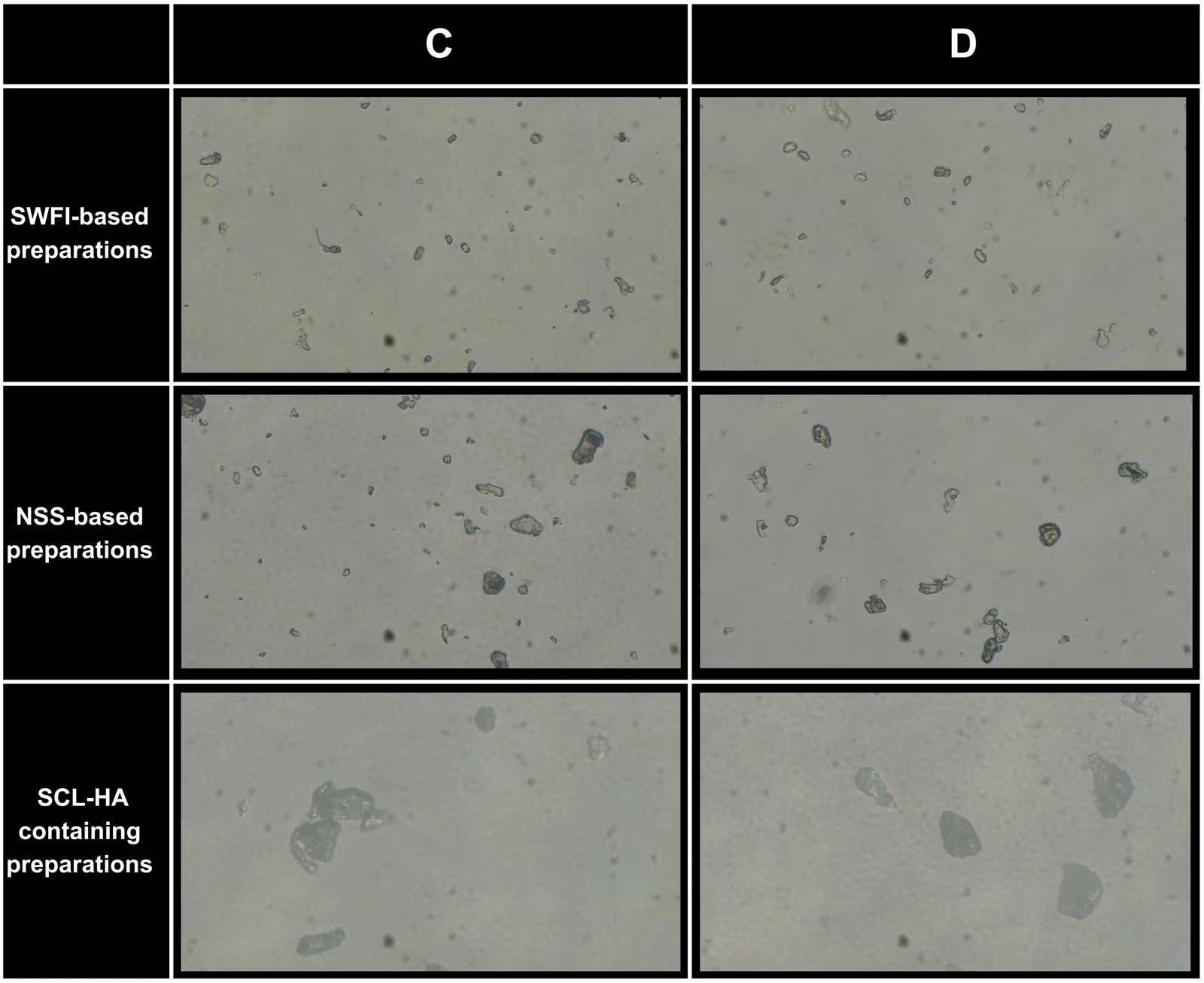
Representative microscopic findings 15 min after final preparation. The figure has been divided into enlarged panels (**A**,**B**) (StiCol Volume) and (**C**,**D**) (StiCol Soft). (**A**,**C**) show the roller/30 min protocol; (**B**,**D**) show the manual/3 h protocol. Rows show representative SWFI-based, NSS-based, and SCL-HA-containing conditions. All images were captured at an original magnification of ×100. Accurate scale bars could not be reconstructed because pixel-to-distance calibration was not retained. Abbreviations: PLLA, poly-L-lactic acid; SWFI, sterile water for injection; NSS, normal saline solution; SCL-HA, slightly cross-linked hyaluronic acid solution.

Representative microscopic images at 15 min showed visually different particle distributions and cluster sizes across conditions (Figure 3). This time point was the earliest microscopic observation and was selected for illustration only. Figure 3 has been enlarged and divided into panels A,B and C,D; all micrographs were captured at an original magnification of ×100. Accurate scale bars could not be added because calibration metadata were unavailable.

At 15 min, SWFI-based image sets generally contained fewer large compact clusters than NSS-based sets. This pattern was most apparent for StiCol Soft and the roller/30 min protocol. NSS-based images contained more frequent clusters and shorter apparent interparticle distances in several fields.

SCL-HA-containing images showed a distinct distribution pattern from NSS-based images, although clusters remained visible. Anesthetic-only conditions showed variable intermediate grades. Given the absence of replicated preparations and standardized random field selection, these observations should not be interpreted as quantified differences between diluents.

Later stored images showed increasing cluster formation in several conditions, particularly StiCol Volume, NSS-based diluents, and the manual/3 h protocol. These serial observations were derived from the same tubes and are descriptive.

### 3.4. Comparison of Commercial Formulations

StiCol Volume generally showed higher visual aggregation grades and more pronounced separation than StiCol Soft across the available image sets. This comparison does not isolate PLLA concentration because the products also differed in particle size, total lyophilized mass, final volume, and potentially excipient proportions and manufacturing characteristics. Accordingly, the observed pattern is reported as a commercial formulation difference. A concentration-specific conclusion would require the same formulation tested at multiple controlled concentrations.

### 3.5. Patterns by Diluent Composition

Across the available image sets, SWFI-based conditions tended to show lower grades than NSS-based conditions. Adding 1 mL of 2% lidocaine to either base vehicle was associated with higher grades in some comparisons, whereas adding adrenaline did not produce a visually consistent additional difference. These observations are descriptive.

NSS-based conditions frequently showed more microscopic clustering and macroscopic vertical separation than SWFI-based conditions. Because ionic strength, pH, and osmolality were not measured, the study cannot identify the cause of this pattern.

SCL-HA-containing and anesthetic-only conditions showed variable intermediate patterns. Their apparent differences from NSS-based preparations require confirmation with replicated preparations and objective measurements.

### 3.6. Comparison of Composite Preparation Protocols

The roller/30 min protocol tended to show lower aggregation grades than the manual/3 h protocol in several comparisons. Because the two protocols differed simultaneously in mechanical action and hydration duration, the observed pattern cannot be assigned to roller mixing itself.

The protocol-level comparison should therefore be interpreted as a comparison of two complete preparation procedures rather than evidence that one mixing method is independently superior.

### 3.7. Time-Dependent Changes

Serial observations showed increasing visual clustering or vertical phase separation in several preparations from 30 min onward (Figure 2 and Supplementary Table S1). The 15 min time point was the earliest microscopic assessment; immediate and 15 min comparisons were available only macroscopically.

The same tubes were followed over time without remixing, so the observations describe behavior under an undisturbed laboratory condition rather than routine clinical handling. They do not establish redispersibility, dose uniformity after agitation, or behavior during syringe withdrawal and needle passage.

At 24 h, several conditions showed more pronounced vertical separation and heterogeneous microscopic fields. Upward accumulation in many gross panels was recorded as creaming/flotation-like behavior, not sedimentation.

No administration-time recommendation can be derived from these observations. The possibility that a shorter delay after final preparation could preserve uniformity is retained only as a testable hypothesis for future studies using standardized remixing, redispersibility, dose-uniformity, and extrusion-force assessments.

## 4. Discussion

This descriptive in vitro pilot study found visually different aggregation grades and vertical phase-separation patterns across two commercial PLLA formulations, eleven diluent conditions, two composite preparation protocols, and serial observation times. The observations are hypothesis-generating because each condition was represented by one preparation, and image acquisition, field selection, and sampling were not fully standardized.

A possible role of ionic strength and CMC behavior has been proposed in prior PDLLA work [8,10,13,14,15], but the present study did not measure ionic strength, pH, osmolality, CMC conformation, or interparticle forces. The PDLLA study therefore supports biological plausibility only; it does not provide mechanistic confirmation for these PLLA products, which differ in stereochemistry, particles, excipients, and manufacturing characteristics.

Lidocaine-containing conditions showed higher visual grades in some comparisons, particularly when combined with NSS. Because stock formulation details, pH, preservatives, and osmolality were not fully characterized, no causal mechanism can be assigned to lidocaine or adrenaline.

SCL-HA-containing conditions showed distinct visual patterns from NSS-based conditions. Any explanation involving buffering, viscosity, or HA-mediated stabilization remains speculative because these properties were not measured. Replicated studies should characterize composition and rheology before attributing a stabilizing effect to SCL-HA.

StiCol Volume generally showed higher grades than StiCol Soft, but the comparison does not isolate concentration. In addition to nominal PLLA concentrations of 16.7 and 8.3 mg/mL, respectively, the products differ in approximate particle size and may differ in excipient proportions and manufacturing features. A concentration–response experiment using a single formulation would be required to test concentration independently.

The roller/30 min and manual/3 h protocols also differed in observed grades. However, the protocol comparison is intrinsically confounded because it changed both the mechanical action and hydration duration. The findings therefore cannot establish that roller mixing is superior to manual shaking. A factorial study matching hydration time and total energy input is needed.

The serial image sets suggest that visual aggregation or vertical phase separation may evolve while an undisturbed preparation stands. Upward accumulation in Figure 2 is more consistent with creaming/flotation-like behavior than settling. Because no redispersibility or dose-uniformity test was performed, the reversibility and practical significance of this separation remain unknown.

The earliest microscopic observation occurred at 15 min. Although some 15 min images appeared more uniform than later images, this does not validate injection within 15 min. Such a window remains an untested hypothesis that must be evaluated after standardized agitation and through measurements of redispersibility, aliquot uniformity, syringe withdrawal, needle passage, and extrusion force.

Uneven particle distribution has been discussed as a possible contributor to papules or nodules after PLLA injection [5,6,7], but the present study did not evaluate tissue distribution, injectability, or clinical adverse events. Visual clusters in vitro therefore cannot be interpreted as evidence of clinical risk or risk reduction.

The study’s principal value is to identify preparation variables and methodological questions for controlled follow-up rather than to define a preferred clinical protocol. Replicated experiments should separate formulation from concentration and mixing mode from hydration duration, and should include calibrated microscopy, randomized field-selection, formal interobserver agreement, redispersibility, particle size distribution, zeta potential, pH, osmolality, rheology, aliquot uniformity, and extrusion testing.

Strengths include the broad matrix of clinically relevant diluent conditions, assessment of two commercial formulations and two preparation protocols, and complementary macroscopic and microscopic documentation across serial time points. The predefined ordinal scale also provided a structured descriptive framework.

The study has important limitations. It used one independent preparation per condition without technical replicates, precluding estimation of within-condition variability and inferential comparison of experimental conditions. Serial observations came from the same tube. The two products differed in more than concentration, and the two preparation protocols differed in both mixing mode and hydration duration. Sampling height, drop volume, and the number and location of microscopic fields were not prospectively standardized; equipment models and calibration metadata were not retained. Evaluators were not fully blinded to the experimental condition, and agreement was evaluated at the condition–time image-set level rather than at the individual-field level. Although exact interobserver agreement was 89.5% and linearly weighted Cohen’s kappa was 0.91, reproducibility across independent preparations could not be assessed. No redispersibility, particle size, zeta potential, pH, osmolality, viscosity, rheology, aliquot uniformity, or extrusion-force measurements were performed. Finally, the undisturbed in vitro observations do not reproduce clinical remixing or injection and cannot establish clinical safety or an administration window.

Future studies should use replicated factorial designs, a single formulation tested at controlled concentrations, matched hydration periods across mixing methods, standardized random sampling, calibrated microscopy with scale bars, and objective physicochemical and injectability outcomes. Ex vivo or in vivo work would then be needed to determine whether any preparation-related difference affects tissue distribution or clinical outcomes.

## 5. Conclusions

In this descriptive pilot study, macroscopic appearance and microscopic aggregation grades varied across two commercial PLLA formulations, diluent conditions, two composite mixing/hydration protocols, and observation time. The product comparison cannot be attributed to concentration alone, and the protocol comparison cannot separate mixing mode from hydration duration. Upward macroscopic accumulation was interpreted as creaming/flotation-like vertical phase separation rather than sedimentation. The findings do not establish irreversibility, dose uniformity, injectability, an administration window, or clinical safety. They provide a basis for replicated, quantitatively characterized studies of reconstitution and redispersibility.

## Figures and Tables

**Table 1:** Component volumes and calculated final lidocaine concentrations.

Diluent Condition	StiCol Volume; Final 9 mL	StiCol Soft; Final 6 mL	Final Lidocaine, Volume/Soft
SWFI	9 mL SWFI	6 mL SWFI	0/0
SWFI + 2% lidocaine	8 mL SWFI + 1 mL 2% lidocaine	5 mL SWFI + 1 mL 2% lidocaine	0.22%/0.33%
SWFI + 2% lidocaine + adrenaline	8 mL SWFI + 1 mL 2% lidocaine/adrenaline stock	5 mL SWFI + 1 mL 2% lidocaine/adrenaline stock	0.22%/0.33%
NSS	9 mL NSS	6 mL NSS	0/0
NSS + 2% lidocaine	8 mL NSS + 1 mL 2% lidocaine	5 mL NSS + 1 mL 2% lidocaine	0.22%/0.33%
NSS + 2% lidocaine + adrenaline	8 mL NSS + 1 mL 2% lidocaine/adrenaline stock	5 mL NSS + 1 mL 2% lidocaine/adrenaline stock	0.22%/0.33%
1% SCL-HA	SCL-HA solution to 9 mL	SCL-HA solution to 6 mL	0/0
SCL-HA + 2% lidocaine	SCL-HA to 8 mL + 1 mL 2% lidocaine	SCL-HA to 5 mL + 1 mL 2% lidocaine	0.22%/0.33%
SCL-HA + 2% lidocaine + adrenaline	SCL-HA to 8 mL + 1 mL 2% lidocaine/adrenaline stock	SCL-HA to 5 mL + 1 mL 2% lidocaine/adrenaline stock	0.22%/0.33%
2% lidocaine alone	9 mL 2% lidocaine	6 mL 2% lidocaine	2.0%/2.0%
2% lidocaine + adrenaline	9 mL 2% lidocaine/adrenaline stock	6 mL 2% lidocaine/adrenaline stock	2.0%/2.0%

Abbreviations: SWFI, sterile water for injection; NSS, normal saline solution; SCL-HA, slightly cross-linked hyaluronic acid. The exact adrenaline stock concentration and preservative content were not retained; pH and osmolality were not measured.

**Table 2:** Semi-quantitative visual aggregation grading system.

Grade	Description
Grade 0	No visible clusters; homogeneous particle distribution with well-separated microspheres
Grade 1	Small clusters present, while most particles remain dispersed
Grade 2	Multiple clusters with reduced interparticle separation
Grade 3	Large compact clusters with marked microscopic heterogeneity

Abbreviation: PLLA, poly-L-lactic acid. This grading system was adapted from previously described semi-quantitative methods used to evaluate dispersion and aggregation characteristics of injectable biomaterials [8].

**Table 3:** Independent evaluator ratings of visual aggregation 15 min after final preparation.

Diluent Condition	Volume Roller/30 min	Volume Manual/3 h	Soft Roller/30 min	Soft Manual/3 h
SWFI	1	1–2	0	0–1
SWFI + 2% lidocaine	1–2	2	1	1
SWFI + 2% lidocaine + adrenaline	1–2	2	1	1
NSS	2–3	3	1–2	2
NSS + 2% lidocaine	3	3	2	2
NSS + 2% lidocaine + adrenaline	3	3	2	2
SCL-HA	1–2	2	1	1
SCL-HA + 2% lidocaine	2	2	1	1–2
SCL-HA + 2% lidocaine + adrenaline	2	2	1	1–2
2% lidocaine	2	2 (2–3)	1	1–2
2% lidocaine + adrenaline	2 (3)	2 (3)	1	1–2

Abbreviations: PLLA, poly-L-lactic acid; SWFI, sterile water for injection; NSS, normal saline solution; SCL-HA, slightly cross-linked hyaluronic acid solution.

## Data Availability

The datasets generated during and/or analyzed during the current study are available from the corresponding author on reasonable request. The data are not publicly available due to restrictions eg privacy or ethical.

## Supplementary Materials

The following supporting information can be downloaded at: https://www.mdpi.com/article/10.3390/polym18172044/s1, Table S1: Semi-quantitative aggregation grading of PLLA suspensions at each time point according to diluent condition, PLLA concentration, and reconstitution method.

## Abbreviations

The following abbreviations are used in this manuscript:

PLLA	Poly-L-lactic acid
SWFI	Sterile water for injection
NSS	Normal saline solution
HA	Hyaluronic acid
SCL-HA	Slightly cross-linked hyaluronic acid solution
SS-A	SS-A additive solution
PDLLA	Poly-D,L-lactic acid
CMC	Carboxymethyl cellulose
min	Minutes
h	Hours

## References

[1] Lee J.C., Lorenc Z.P. (2016). Synthetic Fillers for Facial Rejuvenation. Clin. Plast. Surg..

[2] Fitzgerald R., Bass L.M., Goldberg D.J., Graivier M.H., Lorenc Z.P. (2018). Physiochemical Characteristics of Poly-L-Lactic Acid (PLLA). Aesthet. Surg. J..

[3] Gogolewski S., Jovanovic M., Perren S.M., Dillon J.G., Hughes M.K. (1993). Tissue response and in vivo degradation of selected polyhydroxyacids: Polylactides (PLA), poly(3-hydroxybutyrate) (PHB), and poly(3-hydroxybutyrate-co-3-hydroxyvalerate) (PHB/VA). J. Biomed. Mater. Res..

[4] Vleggaar D., Fitzgerald R., Lorenc Z.P., Andrews J.T., Butterwick K., Comstock J., Hanke C.W., O’Daniel T.G., Palm M.D., Roberts W.E. (2014). Consensus recommendations on the use of injectable poly-L-lactic acid for facial and nonfacial volumization. J. Drugs Dermatol..

[5] Lorenc Z.P., Greene T., Gottschalk R.W. (2016). Injectable Poly-L-Lactic Acid: Understanding Its Use in the Current Era. J. Drugs Dermatol..

[6] Schierle C.F., Casas L.A. (2011). Nonsurgical rejuvenation of the aging face with injectable poly-L-lactic acid for restoration of soft tissue volume. Aesthet. Surg. J..

[7] Fitzgerald R., Vleggaar D. (2011). Facial volume restoration of the aging face with poly-l-lactic acid. Dermatol. Ther..

[8] Chen S.Y., Chen S.T., Lin J.Y., Lin C.Y. (2020). Reconstitution of Injectable Poly-d,l-lactic Acid: Efficacy of Different Diluents and a New Accelerating Method. Plast. Reconstr. Surg. Glob. Open.

[9] CONTACKOREA Inc (2024). StiCol^®^ Product Brochure and Technical Data: Composition, Microsphere and Micelle Technology.

[10] Heinze T., Koschella A. (2005). Carboxymethyl Ethers of Cellulose and Starch—A Review. Macromol. Symp..

[11] Hunter R.J. (2001). Foundations of Colloid Science.

[12] Kubik P., Gruszczyński W., Filipowska M. (2025). Comparative Analysis of Reconstitution and Solubility of Two Poly-L-Lactic Acid Fillers for Medical Applications. Polymers.

[13] Lopez C.G., Colby R.H., Cabral J.T. (2018). Electrostatic and Hydrophobic Interactions in NaCMC Aqueous Solutions: Effect of Degree of Substitution. Macromolecules.

[14] Lopez C.G., Richtering W. (2021). Oscillatory rheology of carboxymethyl cellulose gels: Influence of concentration and pH. Carbohydr. Polym..

[15] Israelachvili J. (2011). Intermolecular and Surface Forces.

