# Supplementary material for: Influence of Reconstitution Methods and Diluents on the Physicochemical Characteristics of Poly-L-Lactic Acid Suspensions: An In Vitro Study
Source: Polymers (Basel). 2026 Aug 23;18(17):2044. doi: 10.3390/polym18172044 (PMC13567586; doi:10.3390/polym18172044)
Supplement: Supplementary file 1 [file polymers-18-02044-s001.zip › polymers-4450240-supplementary.pdf]

Supplementary Table S1. Semi-quantitative aggregation grading of PLLA suspensions at each time point according to diluent condition, PLLA concentration, and reconstitution method

Diluent condition	PLLA concentration	Method	15 min	30 min	45 min	60 min	24 h
SWFI	150 mg	Roller	1	1	1–2	2	2
SWFI	150 mg	Shake	1–2	2	2	2	2–3
SWFI	50 mg	Roller	0	0–1	1	1	1–2
SWFI	50 mg	Shake	0–1	1	1	1–2	2
SWFI + 2% lidocaine	150 mg	Roller	1–2	2	2	2	2–3
SWFI + 2% lidocaine	150 mg	Shake	2	2	2–3	2–3	3
SWFI + 2% lidocaine	50 mg	Roller	1	1	1–2	1–2	2
SWFI + 2% lidocaine	50 mg	Shake	1	1–2	1–2	2	2
SWFI + 2% lidocaine + adrenaline	150 mg	Roller	1–2	2	2	2	2–3
SWFI + 2% lidocaine + adrenaline	150 mg	Shake	2	2	2–3	2–3	3
SWFI + 2% lidocaine + adrenaline	50 mg	Roller	1	1	1–2	1–2	2
SWFI + 2% lidocaine + adrenaline	50 mg	Shake	1	1–2	1–2	2	2
NSS	150 mg	Roller	2–3	3	3	3	3
NSS	150 mg	Shake	3	3	3	3	3

Diluent condition	PLLA concentration	Method	15 min	30 min	45 min	60 min	24 h
NSS	50 mg	Roller	1–2	2	2 (3)	2–3 (3)	3
NSS	50 mg	Shake	2	2–3 (3)	3	3	3
NSS + 2% lidocaine	150 mg	Roller	3	3	3	3	3
NSS + 2% lidocaine	150 mg	Shake	3	3	3	3	3
NSS + 2% lidocaine	50 mg	Roller	2	2–3	3	3	3
NSS + 2% lidocaine	50 mg	Shake	2	3	3	3	3
NSS + 2% lidocaine + adrenaline	150 mg	Roller	3	3	3	3	3
NSS + 2% lidocaine + adrenaline	150 mg	Shake	3	3	3	3	3
NSS + 2% lidocaine + adrenaline	50 mg	Roller	2	2–3	3	3	3
NSS + 2% lidocaine + adrenaline	50 mg	Shake	2	3	3	3	3
SCL-HA	150 mg	Roller	1–2	2	2	2	2–3
SCL-HA	150 mg	Shake	2	2	2	2–3	3
SCL-HA	50 mg	Roller	1	1	1	1–2	2 (2-3)
SCL-HA	50 mg	Shake	1	1–2	1–2	2	2 (2-3)
SCL-HA + 2% lidocaine	150 mg	Roller	2	2	2–3	2–3	3
SCL-HA + 2% lidocaine	150 mg	Shake	2	2–3	2–3	3	3

Diluent condition	PLLA concentration	Method	15 min	30 min	45 min	60 min	24 h
SCL-HA + 2% lidocaine	50 mg	Roller	1	1–2 (1)	1–2	2	2 (3)
SCL-HA + 2% lidocaine	50 mg	Shake	1–2	2 (1-2)	2	2 (2-3)	2–3 (3)
SCL-HA + 2% lidocaine + adrenaline	150 mg	Roller	2	2	2–3	2–3	3
SCL-HA + 2% lidocaine + adrenaline	150 mg	Shake	2	2–3	2–3	3	3
SCL-HA + 2% lidocaine + adrenaline	50 mg	Roller	1	1–2	1–2	2	2 (3)
SCL-HA + 2% lidocaine + adrenaline	50 mg	Shake	1–2	2	2	2 (2-3)	2–3 (3)
2% lidocaine	150 mg	Roller	2	2	2–3	2–3	3
2% lidocaine	150 mg	Shake	2 (2-3)	2–3 (3)	2–3 (3)	3	3
2% lidocaine	50 mg	Roller	1	1–2	1–2	2	2
2% lidocaine	50 mg	Shake	1–2	2	2	2	2–3
2% lidocaine + adrenaline	150 mg	Roller	2 (3)	2 (3)	2–3 (3)	2–3 (3)	3
2% lidocaine + adrenaline	150 mg	Shake	2 (3)	2–3 (3)	2–3 (3)	3	3

Diluent condition	PLLA concentration	Method	15 min	30 min	45 min	60 min	24 h
2% lidocaine + adrenaline	50 mg	Roller	1	1–2	1–2	2	2
2% lidocaine + adrenaline	50 mg	Shake	1–2	2	2	2	2–3

Abbreviations: PLLA, poly-L-lactic acid; SWFI, sterile water for injection; NSS, normal saline solution; SCL-HA, slightly cross-linked hyaluronic acid solution.

Aggregation was graded from 0 to 3, where Grade 0 indicates no visible aggregation with homogeneous particle distribution and Grade 3 indicates severe aggregation with large compact clusters, marked heterogeneity, sedimentation, or phase separation. Scores represent combined interpretation of gross and microscopic findings at each time point. The immediate post-reconstitution time point was not included in this grading matrix because microscopic evaluation was first performed at 15 minutes; immediate gross findings are described in the Results section.
